# miR-181a-5p of MSCs-derived exosomes promote vascular formation and cell proliferation by PTEN/PI3K/AKT axis in HUVECs

**DOI:** 10.1038/s41598-026-44672-5

**Published:** 2026-04-16

**Authors:** Wenxin Shi, Qian Li, Xiaoli Dong, Yanfang Du, Yanlong Fu, Xianghua Huang, Zhongkang Li

**Affiliations:** 1 Department of Obstetrics and Gynecology, Hebei Key Laboratory of Regenerative Medicine of Obstetrics and Gynecology, The Second Hospital of Hebei Medical University, 215 Heping West Road, Shijiazhuang, 050000 Hebei People’s Republic of China; 2 Department of Gynecology, Affiliated Hospital of Chengde Medical University, Chengde, 067000 Hebei People’s Republic of China; 3 Department of Reproductive Medicine, Longyan First Affiliated Hospital of Fujian Medical University, 105 Jiuyi North Road, Longyan, 362300 Fujian People’s Republic of China; 4 Department of Hepatobiliary Surgery, The 980 Hospital of the Joint Service Support Force of the People’s Liberation Army of China, 398 Zhongshan West Road, Shijiazhuang, 050000 Hebei People’s Republic of China

**Keywords:** Mesenchymal stem cell-derived exosomes, miR-181a-5p, PTEN, PI3K/AKT pathway, Vascular formation, Migration, Cell proliferation, Cell biology, Stem cells, Molecular medicine

## Abstract

Our previous studies have demonstrated that exosomes play a crucial role in promoting vaginal tissue reconstruction in rats. The present study aims to elucidate the molecular mechanisms through which human umbilical cord mesenchymal stem cell-derived exosomes (hUMSC-Exos), which carry microRNA-181a-5p (miR-181a-5p), promote vascularization and tissue regeneration, with particular focus on the involvement of the PTEN/PI3K/AKT signaling pathway. Human umbilical vein endothelial cells (HUVECs) served as a model for studying angiogenesis and cell proliferation, and the expression levels of miR-181a-5p, PTEN, phospho-PI3K (p-PI3K), and phospho-AKT (p-AKT) were analyzed. HUVECs were transfected with PTEN overexpression vector or a negative control vector, then treated with exosomes derived from mesenchymal stem cells (MSCs) transfected with either a miR-181a-5p mimic or an inhibitor. Cell proliferation and migration were assessed using the Cell Counting Kit-8 and scratch assay, respectively, while cell invasion was evaluated via Transwell assay. The StarBase tool was employed to predict binding sites between miR-181a-5p and its target gene, the phosphatase and tensin homolog (PTEN). This interaction was subsequently validated using a dual-luciferase reporter assay. In HUVECs, elevated miR-181a-5p levels were positively correlate with reduced PTEN expression. In vitro experiments demonstrate that hUMSC-Exos enhance HUVEC migration, proliferation, and tube formation. Furthermore, overexpression of PTEN partially counteracted these miR-181a-5p-mediated effects. Our findings indicate that hUMSC-Exos contain miR-181a-5p, which may enhance tube formation and proliferation in HUVECs by regulating PTEN expression, thereby influencing the PI3K/AKT pathway.

**Supplementary Information:**

The online version contains supplementary material available at 10.1038/s41598-026-44672-5.

## Introduction

Tissue regeneration is a complex process involving cell migration, proliferation, angiogenesis, and other biological functions^1^. Angiogenesis plays a pivotal role in this process. Current therapeutic strategies to promote angiogenesis and tissue regeneration include the use of cytokines, growth factors, as well as cell therapies. Among these, stem cell therapy has emerged as a prominent approach for tissue regeneration and repair^2,3^. MSCs have been demonstrated to accelerate wound healing through their self-renewal capacity^4^ and reduce risks associated with stem cell therapy via paracrine effects^5^. These mechanisms are considered key factors in promoting tissue repair^6^. Exosomes—nanoscale extracellular vesicles ranging from 30 to 100 nm in diameter—have garnered significant attention for their role in intercellular communication^7,8^. These vesicles facilitate the intercellular transfer of regulatory molecules and are increasingly recognized as important mediators in both physiological and pathological processes, holding great potential applications in disease diagnosis and treatment^9^. Exosomal microRNAs (miRNAs) exhibit high stability due to their encapsulation within vesicles, which protects them from degradation by RNases^10^. These miRNAs, upon delivery into recipient cells, can regulate gene expression and modulate intracellular signaling pathways, thereby influencing diverse biological processes such as cell proliferation, metastasis, and therapeutic resistance^11^. hUCMSC-Exos represent an acellular, multifunctional, and highly effective next-generation therapeutic strategy. Their unique advantages stem from their biological origin, safety profile, and bioactivity, which collectively enable the modulation of multiple disease-related pathways. By carrying bioactive mediators, exosomes can reprogram recipient cells and promote angiogenesis, immunomodulation, and tissue regeneration^12,13^. Recent studies have also demonstrated that miRNAs within exosomes can regulate macrophage polarization by modulating oxidative stress and redox signaling^14–16^. As important biological carriers, exosomes hold significant application potential in regenerative medicine and angiogenesis. However, their specific molecular mechanisms remain incompletely understood and warrant further investigation.

Exosomes are extracellular vesicles produced through paracrine effects that facilitate the intercellular transfer of various miRNAs, mRNAs, and proteins^17^. In animal models, their role in soft tissue repair and regeneration has been well demonstrated^18–21^. miRNAs are short non-coding RNA molecules, typically 20–25 nucleotides in length^22^, that regulate diverse biological processes by binding to target mRNAs to inhibit translation or promote their degradation^23^. Among these, the miR-181 family is widely expressed across multiple tissues and cell types—particularly in the immune, nervous, cardiovascular, and musculoskeletal systems^24^. In humans, this family comprises four mature 5p isoforms: miR-181a-5p, miR-181b-5p, miR-181c-5p, and miR-181d-5p, encoded by three gene clusters located on chromosomes 1, 9, and 19^25^. All members possess a conserved 5′ seed sequence (ACAUUCA), reflecting high evolutionary conservation^26^. Functionally, miR-181 family members participate in diverse biological processes^24,27^, ranging from early embryonic development to adult tissue homeostasis, with a particularly prominent role in vasculogenesis and angiogenesis^28–30^. Importantly, different members of the miR-181 family exhibit distinct and sometimes specific functions in the regulation of angiogenesis. Specifically, miR-181a-5p is encoded in the human genome on chromosomes 1 and 9, and its mature sequence is highly conserved across species. The mature form of miR-181a-5p is a single-stranded molecule with the sequence “AACAUUCAACGCUGUCGGUGAGU”^15,20^. Functionally, it post-transcriptionally regulates gene expression by binding to the 3’ untranslated region (3'UTR) of target mRNAs, leading to either transcript degradation or translational inhibition^31^.

As a conserved miRNA, miR-181a-5p participates in multiple pathological processes, including angiogenesis, inflammatory responses, and obesity, with its expression levels also being regulated by various signaling pathways^32,33^. For instance, miR-181a-5p has been demonstrated to promote colorectal cancer metastasis and cell proliferation by suppressing PTEN, a well-characterized tumor suppressor gene^34^. Multiple studies have confirmed that miR-181a-5p directly binds to the 3′UTR of PTEN mRNA, downregulating its expression and thereby activating the phospho (p)-AKT pathway^35,36^.

As a well-established tumor suppressor, PTEN plays a crucial role in multiple cellular processes^37^. Indeed, PTEN has been demonstrated to physically interact with multiple critical cellular structures, including DNA replication forks, DNA damage repair sites, nucleosomes, and the mitotic spindle^37^. Through conditional interactions with distinct mechanisms involved in DNA replication, chromosome segregation, and chromatin remodeling, PTEN participates in diverse signaling pathways. Increasing evidence indicates that the PTEN/PI3K/AKT axis plays a crucial role in angiogenesis^38–40^, and it has been demonstrated that activation of the PI3K/AKT pathway promotes vascular formation^41–43^. Multiple studies have confirmed that miR-181a-5p directly targets PTEN. For instance, in prostate cancer, miR-181a-5p induces PTEN degradation and activates the PI3K/AKT/mTOR signaling pathway, accelerating tumor progression^44^. Similarly, in breast cancer research, the miR-181a-5p/PTEN axis has been demonstrated to regulate cell proliferation, invasion, and glycolysis^45^. Based on these findings, this study aimed to investigate whether miR-181a-5p from hUMSC-Exos promotes angiogenesis and cell proliferation via the PTEN/PI3K/AKT pathway. Herein, hUCMSC-Exos refers to exosomes derived from hUCMSCs.

## Materials and methods

### The sequencing of exosome and bioinformatic analysis

Total exosomal RNA, including miRNA, was isolated from human umbilical cord mesenchymal stem cells (hUCMSCs; Shanghai ZhongQiao Xin Zhou Biotechnology Co., Ltd., Shanghai, China; Cat# DF-GMP-ZB09BA). Small RNA sequencing libraries were prepared using the TruSeq Small RNA Library Prep Kit (Illumina, Inc.) and sequenced on the Illumina HiSeq 2000/2500 platform with 50 bp single-end reads. Following sequencing of hUCMSC-Exos, the 60 most abundant miRNAs were selected for further analysis. Putative target genes of these miRNAs were predicted using three public databases: StarBase (http://starbase.sysu.edu.cn), miRWalk (http://mirwalk.umm.uni-heidelberg.de), and MiRTarBase (https://mirtarbase.cuhk.edu.cn). To investigate the potential functions of the predicted target genes, Gene Ontology (GO) terms, including biological processes (BP), cellular components (CC), and molecular functions (MF), as well as Kyoto Encyclopedia of Genes and Genomes (KEGG) pathway enrichment analyses were performed. The gene ratio was defined as the number of genes annotated to a given term or pathway relative to the total number of annotated genes in the background set. To further investigate miRNA–mRNA–pathway interactions involving hub genes, protein–protein interaction (PPI) networks were constructed using the Search Tool for the Retrieval of Interacting Genes (STRING) database (https://www.string-db.org) with a medium confidence score threshold of 0.4. The resulting PPI networks were visualized using Cytoscape (version 3.8.0), and genes with high connectivity were identified as hub genes. Finally, an integrated miRNA–mRNA–pathway regulatory network was constructed to illustrate the potential regulatory mechanisms.

### Isolation and fluorescent labeling of exosomes

Exosomes were isolated according to a previously described protocol^46,47^. In brief, conditioned medium was collected from hUCMSCs at passages 4–8 and centrifuged at 2000 × g for 15 min at 4 °C to remove intact cells. The supernatant was subsequently centrifuged at 10,000 × g for 30 min at 4 °C to eliminate cellular debris. The resulting supernatant was then subjected to ultracentrifugation at 120,000 × g for 70 min at 4 °C. The pellet was washed with phosphate-buffered saline (PBS; Servicebio, China) and ultracentrifuged again under identical conditions. The final exosome pellet was resuspended in pre‑chilled PBS at a concentration of 3 mg/mL (equivalent to 1.107 × 1011 particles/mL). The exosomes used in this study were consistent with those characterized in our previous reports^46,47^.

Exosomes were labeled with PKH67, a lipophilic fluorescent dye (Beijing Solarbio Science). Briefly, 50 µL of exosomes were resuspended in PBS and mixed with 250 µL of Diluent C. In parallel, 4 µL of PKH67 was added to 300 µL of Diluent C. The two mixtures were then combined and incubated at room temperature for 5 min. The labeling reaction was terminated by adding 600 µL of 1% bovine serum albumin (BSA) and incubated for 1 min. The total volume was adjusted to 20 mL. Labeled exosomes were centrifuged at 100,000 g for 70 min, and the pellet was resuspended in 1 mL PBS. HUVECs were incubated with 200 µL of labeled exosomes for 12 h. After washing with PBS, cells were fixed and stained with 4′,6‑diamidino‑2‑phenylindole (DAPI). Exosome internalization by HUVECs was examined using a confocal microscope.

### MSCs modified by miR-181a-5p

The miR‑181a‑5p mimic, inhibitor, and their corresponding negative control (NC) sequences were obtained from GenePharma (Shanghai, China). Before lentiviral transduction, hUCMSCs at an appropriate passage were seeded at a density of approximately 1 × 105 cells per well and cultured overnight. Culture supernatants were collected for hUMSC‑Exos isolation at 2 days post‑transduction. The isolated exosomes were divided into four groups and transfected with mimic‑NC, miR‑181a‑5p mimic, inhibitor‑NC, and miR‑181a‑5p inhibitor. The exosome groups were designated as follows: MSC‑Exo‑mimic‑NC (mimic‑NC), MSC‑Exo‑miR‑181a‑5p‑mimic (mimic), MSC‑Exo‑inhibitor‑NC (inhibitor‑NC), and MSC‑Exo‑miR‑181a‑5p‑inhibitor (inhibitor). The efficiency of miR‑181a‑5p overexpression or inhibition was verified by reverse transcription quantitative polymerase chain reaction (RT‑qPCR).

### Cell transfection

HUVECs (iCell-h110, iCell Bioscience Inc.) were cultured in exosome‑free endothelial cell basal medium (iCell Bioscience Inc.) supplemented with 5% FBS and 1% endothelial cell culture supplement (both obtained from iCell Bioscience Inc.), and cultured in a humidified incubator at 37 °C with 5% CO₂. Cell transfection was performed using Lipofectamine 3000 reagent (Invitrogen; Thermo Fisher Scientific, Inc.) according to the manufacturer’s instructions. Short hairpin RNA targeting PTEN (sh‑PTEN) and non‑targeting control (sh‑NC) were obtained from GenePharma and used at a concentration of 50 nM for knockdown assays. In addition, the PTEN overexpression plasmid pcDNA3.2‑PTEN and the corresponding empty vector control (pcDNA3.2‑NC) were also employed.

### Vasculogenesis assay

Pre‑cooled 96‑well plates were coated with 100 μL Matrigel (Corning Inc.) and incubated at 37 °C for 30 min to allow polymerization. HUVECs were seeded at a density of 3.0 × 104 cells/cm2 and treated with exosomes at various concentrations (0, 25, 50, and 100 μg/mL) or under different experimental conditions (i.e., exosomes with modulated miR‑181a‑5p expression or cells with altered PTEN expression) for 4 h. Tube formation was visualized and imaged using an inverted light microscope (Imager.D2; Zeiss AG).

### Cell counting kit-8 (CCK-8) assay for evaluation of cell proliferation

The proliferative effect of exosomes on HUVECs was assessed by the Cell Counting Kit‑8 (CCK‑8; Wuhan Servicebio Technology Co., Ltd.). HUVECs were seeded into 96‑well plates at a density of 5,000 cells per well in serum‑free medium, with three replicate wells per group. Cells were treated with exosomes at various concentrations (0, 25, 50, and 100 μg/mL) or under different experimental conditions (i.e., exosomes with modulated miR‑181a‑5p expression or cells with altered PTEN expression), and incubated for 1, 2, or 3 days. Subsequently, 10 μL of CCK‑8 reagent was added to each well. The absorbance at 450 nm was detected using a microplate reader (Synergy‑H1; BioTek Instruments, Inc.), and the data were recorded for further analysis.

### Detection of cell migration by the scratch test

HUVECs were seeded into 6‑well plates at a density of 5.0 × 104 cells/cm2 and cultured for 24 h under standard conditions (37 °C, 5% CO₂). Following two washes with PBS, a linear wound was scratched in the cell monolayer across each well using a sterile 200 μL pipette tip. The cells were then treated with exosomes at various concentrations (0, 25, 50, and 100 μg/mL) or under different experimental conditions (i.e., exosomes with modulated miR‑181a‑5p expression or cells with altered PTEN expression) and further incubated for 6, 12, or 24 h. Wound areas were imaged at 0, 6, 12, and 24 h using an inverted light microscope (Imager.D2; Zeiss AG).

### Transwell migration/invasion assay

The Transwell assay was employed to evaluate the invasive capacity of HUVECs following treatment with various concentrations of exosomes. Cells in the logarithmic growth phase were harvested, washed, and resuspended in serum‑free medium. A total of 2 × 104 HUVECs were seeded into the upper chamber of 24‑well Transwell inserts (8 μm pore size; Corning Inc.). The lower chamber was filled with complete medium supplemented with 10% FBS as a chemoattractant. Plates were incubated at 37 °C for 24 h. After incubation, non‑invading cells on the upper membrane surface were carefully removed with a cotton swab. Invasive cells on the lower surface were fixed with 4% paraformaldehyde for 30 min, washed with PBS, and stained with Giemsa solution at room temperature for 20 min. Following several PBS washes, stained cells were observed and imaged using an inverted optical microscope (Imager.D2; Zeiss AG).

### RT-qPCR

Total RNA was extracted using TRIzol reagent (Solarbio Science), and reverse transcription was performed in accordance with the manufacturer’s instructions (Thermo Fisher Scientific, USA). Primers were synthesized by GenePharma, and the sequences are listed in Table 1. cDNA (10 μL) was amplified using SYBR PCR Master Mix (Thermo Fisher Scientific, USA) on a quantitative real‑time PCR system. The relative expression levels of miR‑181a‑5p and PTEN mRNA were quantified using the 2 − ΔΔCt method, with GAPDH and U6 employed as internal controls.

**Table 1:** Primer sequence (universal reverse primer was used).

Primers	Sequences (5′-3′)
miR-181a-5p-F	CGAACATTCAACGCTGTCG
miR-126a-3p-F	CGCGTCGTACCGTGAGTAAT
miR-103a-5p-F	GCGGGCTTCTTTACAGTGCT
miR-199a-5p-F	CGCGCCCAGTGTTCAGACTAC
miR-148a-3p-F	GCGCGTCAGTGCACTACAGAA
miR-29a-3p-F	CGCGTAGCACCATCTGAAAT
universal reverse primer	AGTGCAGGGTCCGAGGTATT
U6-F	CTCGCTTCGGCAGCACATATACT
U6-R	ACGCTTCACGAATTTGCGTGTC
PTEN-F	AAGACCATAACCCACCACAGC
PTEN-R	ACCAGTTCGTCCCTTTCCAG
sh-PTEN-F	CCGGGCTAGAACTTATCAAACCCTTCTCGAGAAGGGTTTGATAAGTTCTAGCTTTTT
sh-PTEN-R	AATTAAAAAGCTAGAACTTATCAAACCCTTCTCGAGAAGGGTTTGATAAGTTCTAGC
sh-NC-F	CCGGTTCTCCGAACGTGTCACGTTTCTCGAGAAACGTGACACGTTCGGAGAATTTTT
sh-NC-R	AATTAAAAATTCTCCGAACGTGTCACGTTTCTCGAGAAACGTGACACGTTCGGAGAA
GAPDH-F	AATGGGCAGCCGTTAGGAAA
GAPDH-R	GCGCCCAATACGACCAAATC

#### Western blot analysis

Protein was extracted using radio immunoprecipitation assay (RIPA) lysis buffer (Solarbio Science) supplemented with protease inhibitor, and quantified with the Bicinchoninic Acid (BCA) assay kit (Thermo Fisher Scientific, Inc.). After adding appropriate loading buffer, protein samples were loaded into each lane. Proteins were separated by 10% sodium dodecyl sulfate polyacrylamide gel electrophoresis (SDS‑PAGE; Biotides) at a constant current of 200 mA and subsequently transferred onto a polyvinylidene difluoride (PVDF) membrane (0.45 μm; EMD Millipore). The membrane was blocked with 5% skimmed milk for 1 h. Primary antibody incubation was performed overnight at 4 °C at the following dilutions: PTEN (1:750, Zen‑Bioscience), PI3K (1:1200, Zen‑Bioscience), p‑PI3K (1:1000, Zen‑Bioscience), AKT (1:1200, Zen‑Bioscience), p‑AKT (1:1000, Zen‑Bioscience), and GAPDH (1:3000, Zen‑Bioscience). After three washes with Tris-buffered saline with Tween 20 (TBST; Solarbio), the membranes were incubated with HRP‑conjugated secondary antibody (M5 Goat Anti‑Rabbit IgG‑HRP; 1:10,000; Zen‑Bioscience) for 1 h. Following thorough TBST washes, chemiluminescent substrate (Bio‑Rad) was applied uniformly onto the membrane. Protein band signals were visualized and quantified using ImageJ software.

#### Dual-luciferase gene assay

The targeting relationship between miR‑181a‑5p and PTEN was predicted using StarBase. Reporter plasmids containing the wild‑type (WT) or mutant (MUT) 3′‑untranslated region (3′‑UTR) of PTEN were constructed and cloned into the pmirGLO vector (GenePharma). The constructed plasmids were then transfected into 293T cells (Shanghai Zhong Qiao Xin Zhou Biotechnology Co., Ltd.). At 24 h post‑transfection, Renilla and firefly luciferase activities were measured using a dual‑luciferase reporter assay system (Promega Corporation) according to the manufacturer’s instructions. Relative luciferase activity was calculated as the ratio of firefly luciferase activity to Renilla luciferase activity.

#### Statistical analysis

The data were analyzed using SPSS 20.0 (IBM Corp.) and GraphPad Prism 7.0 (GraphPad Software, Inc.). All results are presented as mean ± SD. Statistical comparisons between groups were performed using Student’s t-test or one-way analysis of variance, as appropriate. A p-value of less than 0.05 was considered statistically significant.

## Results

### Integrated bioinformatics analysis identifies a candidate miRNA-pathway axis regulating angiogenesis

To elucidate the molecular mechanisms by which hUMSC-Exos promotes angiogenesis, a bioinformatic strategy was employed. Analysis of exosomal miRNA sequencing data, combined with target gene prediction and network construction, revealed that miR-181a-5p targets multiple genes enriched in angiogenesis-related pathways.

**Fig. 1:**
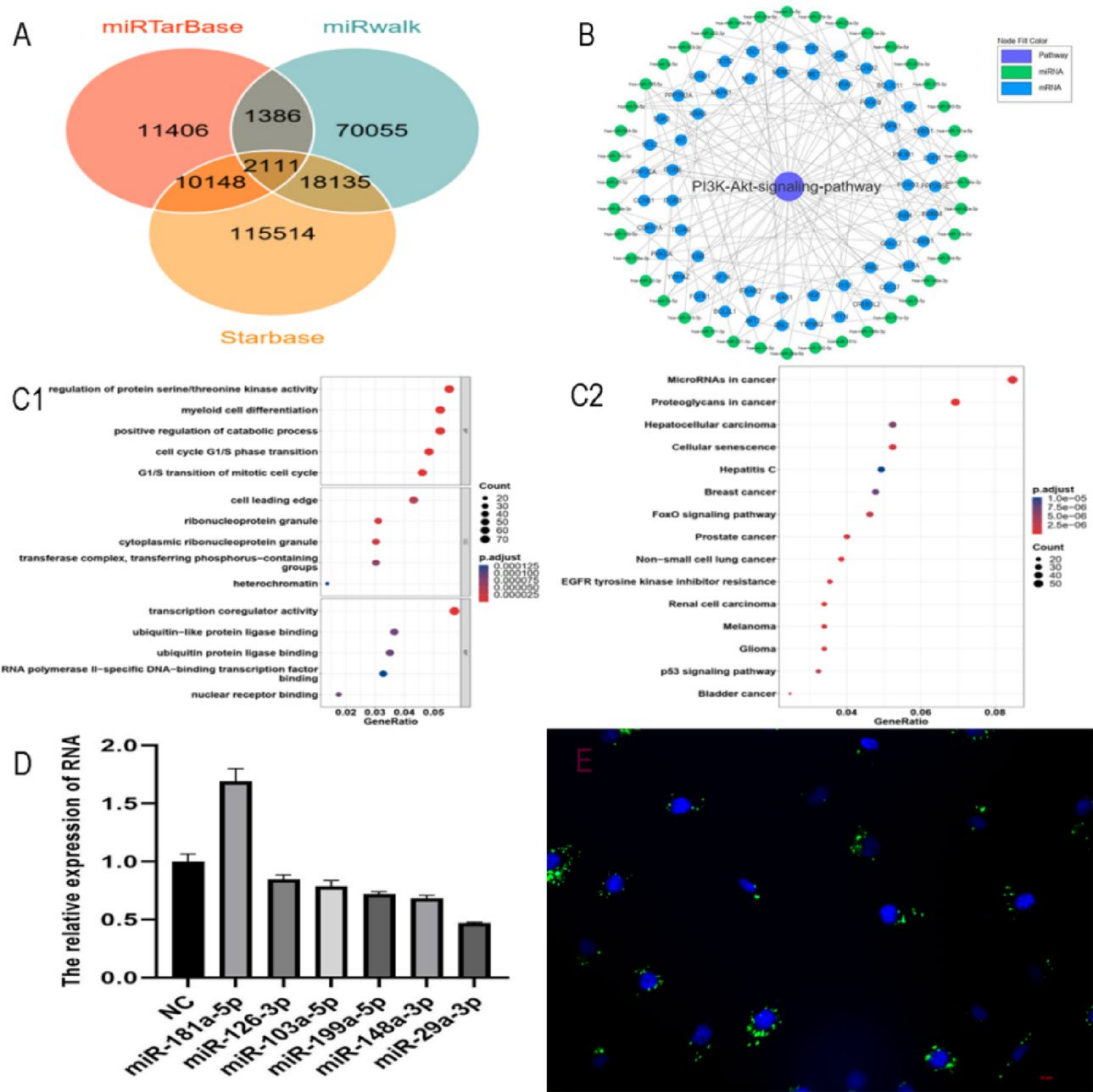
Bioinformatic analysis and cellular uptake of hUCMSC-Exos. (**A**) Venn diagram illustrating the overlap of predicted target genes from multiple miRNA databases. (**B**) Integrated miRNA-mRNA-pathway interaction network. (**C**) Functional enrichment analysis of the predicted target genes. (**C1**) Bar plot of the top five significantly enriched Gene Ontology (GO) terms from each category (Biological Process, Molecular Function, Cellular Component), ranked by adjusted p-value. The y-axis shows the specific GO terms, and the x-axis indicates the Gene Ratio (the proportion of target genes annotated to a given term). (**C2**) Bubble plot of significantly enriched Kyoto Encyclopedia of Genes and Genomes (KEGG) pathways. The y-axis lists the pathway names, and the x-axis represents the Gene Ratio (defined as the number of target genes mapped to a pathway divided by the total number of background genes). The size of each bubble corresponds to the Count of target genes, and the color gradient indicates the statistical significance based on the –log^10^(adjusted p-value). (**D**) The relative expression level of miR-181a-5p in hUCMSC-Exos was quantified by qRT-PCR. (**E**) Confocal microscopy images demonstrate the internalization of PKH67-labeled exosomes (green) by HUVECs in vitro. Cell nuclei were counterstained with DAPI (blue). Scale bar: 20 µm.

Based on miRNA sequencing data of hUMSC‑Exos obtained in our previous study, three databases (Starbase, miRwalk, and MiRTarBase) were used to predict target mRNAs of highly expressed miRNAs. Venn diagram analysis identified 2,111 overlapping miRNA‑mRNA interaction pairs, including 1,364 mRNAs and 55 miRNAs (Fig. 1A). Genes involved in the PI3K/AKT signaling pathway were selected to construct a miRNA‑mRNA‑pathway network (Fig. 1B). The PI3K‑AKT signaling pathway was significantly enriched (*p* < 0.05); 54 genes mapped to this pathway were subjected to further analysis. By integrating these 54 pathway‑related genes with the predicted miRNA‑mRNA pairs, a regulatory network consisting of 40 miRNAs, 54 mRNAs, and the PI3K‑AKT pathway was established (Fig. 1B; supplementary file).GO and KEGG enrichment analyses were performed on the overlapping mRNAs. A total of 883 GO terms and 86 KEGG pathways were significantly enriched (supplementary files: GO-enrich.csv, KEGG-enrich.csv), and the top terms were displayed as bubble plots (Fig. 1C). Subsequently, miRNA expression in exosomes was verified by PCR. The results showed that miR‑181a‑5p exhibited a relatively high expression level compared with other detected miRNAs (Fig. 1D). Furthermore, we confirmed that the exosomes could be efficiently internalized by HUVECs (Fig. 1E).

### Exosomes could promote the angiopoiesis, migratory and proliferative capacity of HUVECs

Bioinformatics analysis strongly indicated that exosomal cargo is involved in angiogenesis-related processes. The effects of hUMSC-Exos were subsequently investigated. HUVECs treated with isolated exosomes exhibited significantly enhanced angiogenesis, migration, and proliferation.

**Fig. 2:**
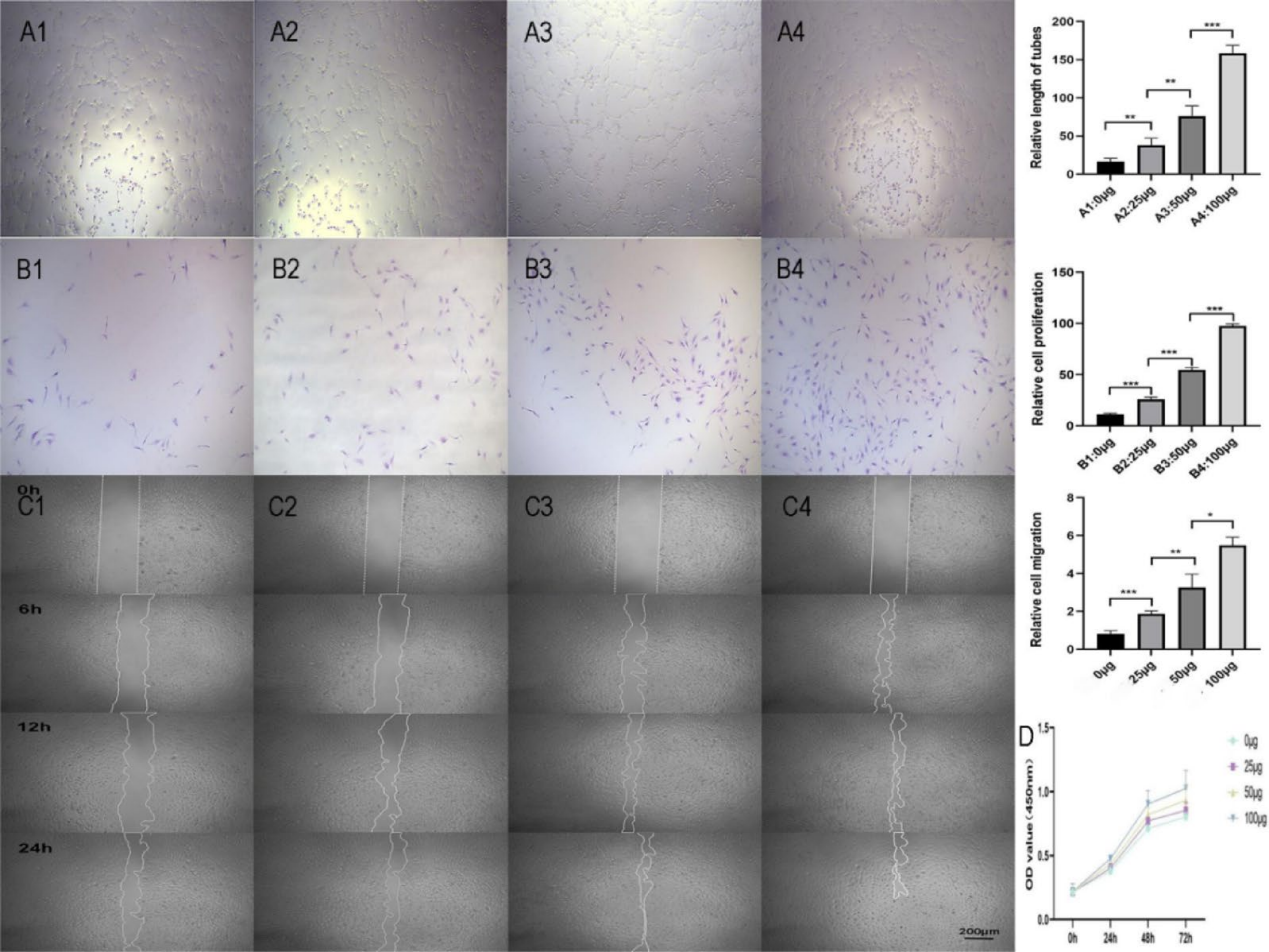
Biological functions of exosomes on HUVECs. (**A**) The image of angiogenesis; (the amount of exosomes in A1:0µg; A2:25µg; A3:50µg; A4:100µg). (**B**) The image of transwell; (the amount of exosomes in B1:0µg; B2:25µg; B3:50µg; B4:100µg); (**C**) The results of scratch test (the amount of exosomes in C1:0µg; C2:25µg; C3:50µg; C4:100µg); (**D**) The results of cell proliferation of HUVECs. Data are presented as the mean ± SD from three independent experiments. Scale bar: 200 µm. (*p < 0.05, **p < 0.01, ***p < 0.001).

Exosomes at higher concentrations significantly enhanced tube formation compared to the lower concentration group (Fig. 2A). In the Transwell assay, HUVECs treated with higher concentrations of exosomes exhibited enhanced invasive ability (Fig. 2B). The scratch assay further confirmed that wound healing improved progressively with increasing exosome concentrations (Fig. 2C). Additionally, results from the CCK-8 assay indicated that exosomes markedly promoted the proliferation of HUVECs in a concentration-dependent manner (Fig. 2D).

### miR-181a-5p regulates the angiogenesis, proliferation, invasiveness and migration capability of HUVECs

**Fig. 3:**
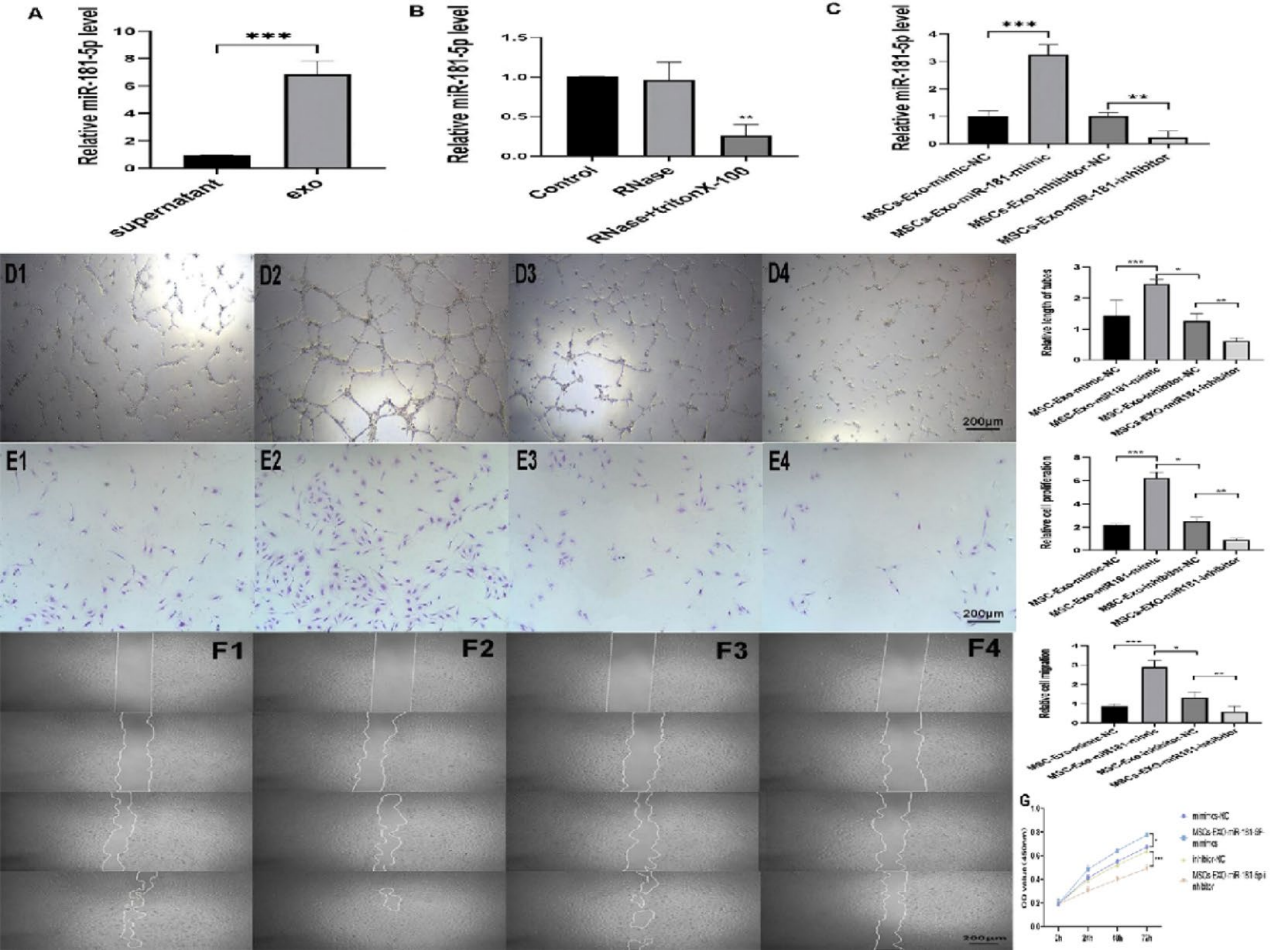
Functional role of exosomal miR-181a-5p in regulating HUVEC behavior. (**A**) miR-181a-5p is enriched in hUCMSC-Exos. (**B**) Exosomal miR-181a-5p is protected within the vesicle membrane. Expression of miR-181a-5p in exosomes treated with RNase A alone or with RNase A combined with Triton X-100 lysis, compared to untreated control. (**C**) Efficient modulation of miR-181a-5p in HUVECs. qRT-PCR was used to validate the transfection efficiency following transfection with miR-181a-5p mimic, mimic negative control (NC), inhibitor, or inhibitor NC. (**D**) Representative images of capillary-like structures. Scale bar: 200 µm. (**E**) Representative images from Transwell assays. Scale bar: 100 µm. (**F**) Representative images from scratch wound healing assays at 0 and 24 h. Dashed lines indicate initial wound edges. Scale bar: 200 µm. (**G**) Cell proliferation assay. Growth curves of HUVECs measured by CCK-8 assay at 24, 48, and 72 h post-transfection. Data are mean ± SD (*n* = 6). *p < 0.05,** p < 0.01. ***p < 0.001. (For **D**–**G**′: Groups are: 1, mimic-NC; 2, miR-181a-5p mimic; 3, inhibitor-NC; 4, miR-181a-5p inhibitor.)

To verify the key regulatory role of miR‑181a‑5p predicted by bioinformatic network analysis, we altered its intracellular expression in HUVECs. Transfection with a miR‑181a‑5p mimic markedly enhanced tube formation (Fig. 3), recapitulating the pro‑angiogenic effect of hUCMSC‑Exos and supporting our in silico prediction.

The expression level of miR‑181a‑5p was determined by RT‑qPCR in exosomes and the corresponding exosome‑depleted supernatant. miR‑181a‑5p was found to be significantly enriched in hUCMSC‑Exos (Fig. 3A). This enrichment was markedly abolished following treatment with Triton X‑100 and RNase (Fig. 3B). Furthermore, transfection with the miR‑181a‑5p mimic and inhibitor significantly upregulated and downregulated its expression, respectively, compared with the negative control (NC) group (Fig. 3C).

Functional experiments demonstrated that overexpression of miR-181a-5p promoted tube formation in HUVECs relative to the mimic-NC group (Fig. 3D). Conversely, inhibition of miR-181a-5p significantly suppressed the invasive and migratory abilities of HUVECs compared with the inhibitor-NC group, as determined by Transwell and scratch wound assays (Fig. 3E, F). Accordingly, the miR-181a-5p mimic enhanced cell invasion and migration, whereas the miR-181a-5p inhibitor exerted the opposite effects.

A consistent tendency was observed in cell proliferation. The CCK‑8 assay showed that the OD_450_ value was elevated in the mimic group but reduced in the inhibitor group (Fig. 3G). Collectively, these results demonstrate that miR‑181a‑5p encapsulated in hUCMSC‑Exos facilitates the proliferation, migration, and angiogenesis of HUVECs.

PTEN counteracts the promsing effect of exosomal miR-181a-5p on HUVECs.

**Fig. 4:**
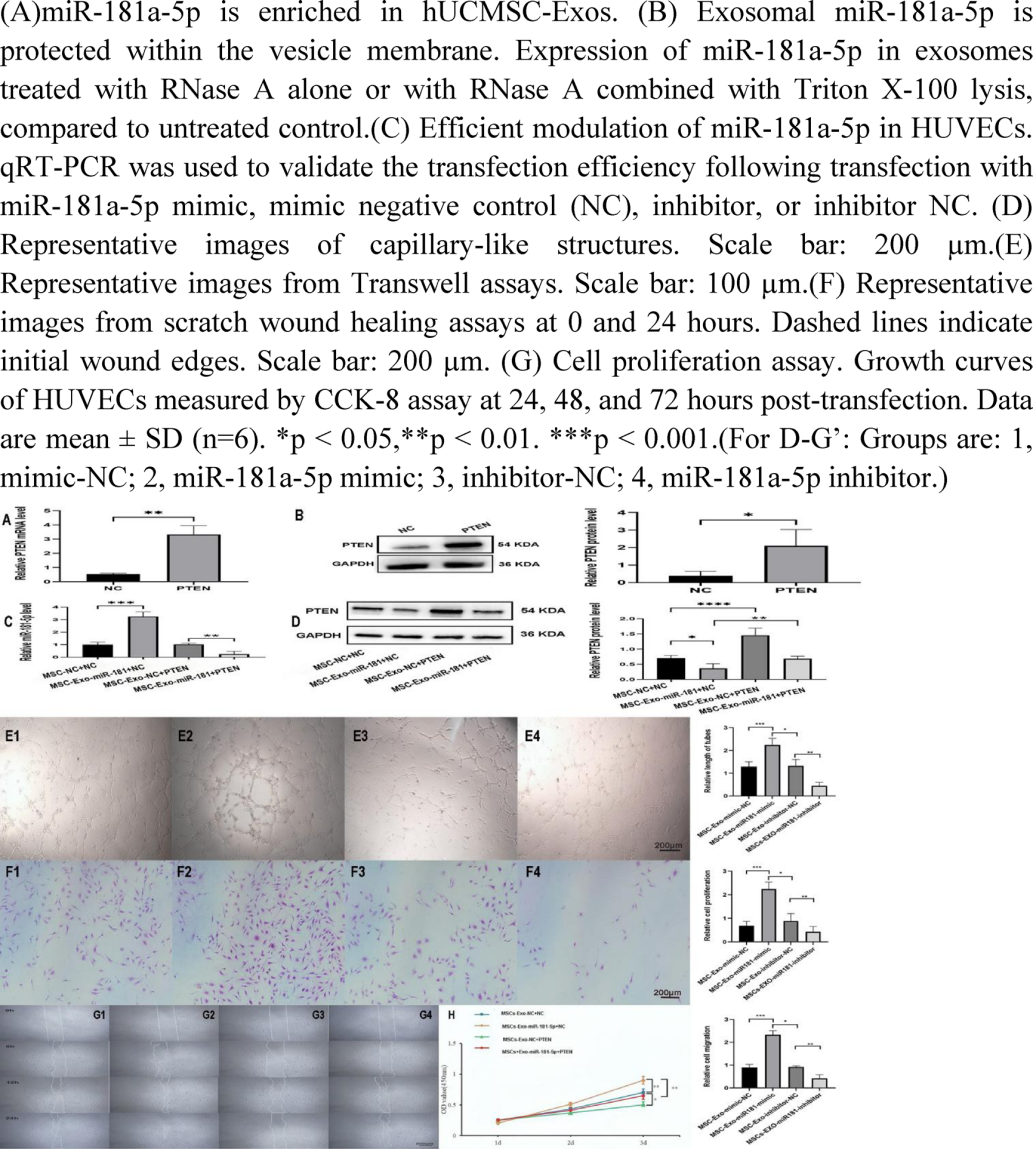
The expression and biological functions of PTEN on HUVECs. (**A**) and (**B**) The expression level of PTEN in HUVECs transfected with pcDNA3.1 or pcDNA3.1-PTEN; (**C**) and (**D**) The expression level of PTEN in HUVECs after transfected with PTEN, and added miR-181a-5p mimic or mimic-NC; (**E**) The image of angiogenesis; (**E1**: MSC-NC+NC; **E2**: MSC-Exo-miR-181a-5p+ NC;E3:MSC-Exo-NC+PTEN;E4:MSC-Exo-miR-181a-5p+PTEN); (**F**) The image of transwell; (**F1**: MSC-NC+NC; **F2**: MSC-Exo-miR-181a-5p+NC; **F3**: MSC-Exo-NC+PTEN; **F4**: MSC-Exo-miR-181a-5p+PTEN); (**G**) The results of scratch test (**G1**: MSC-NC+NC; **G2**: MSCs-Exo-miR-181a-5p+NC; **G3**: MSC-Exo-NC+PTEN; **G4**: MSC-Exo-miR-181a-5p+PTEN); (**H**) The results of cell proliferation of HUVECs (*p < 0.05; ** p < 0.01; *** p < 0.001.**** p < 0.0001).

The transfection efficiency of PTEN was verified by RT-qPCR and western blot analyses (Fig. 4A, B). PTEN expression was the lowest in the MSC-Exo-miR-181a-5p+NC group, intermediate in the NC+NC group, and the highest in the MSC-Exo-NC+PTEN group. PTEN expression was successfully rescued in the MSC-Exo-miR-181a-5p+PTEN group compared with the MSC-Exo-miR-181a-5p+NC group (Fig. 4C, D).

In functional experiments, the tube formation assay demonstrated that the MSC-Exo-miR-181a-5p+NC group significantly promoted HUVEC tube formation compared to the MSC-Exo-NC+NC group, whereas this pro-angiogenic effect was markedly reduced in the MSC-Exo-NC+PTEN group (Fig. 4E). Similarly, PTEN overexpression effectively reversed the enhanced invasive and migratory capacities induced by exosomal miR-181a-5p (Fig. 4F, G).

Consistent with these observations, CCK-8 assay results showed that the OD450 values decreased sequentially from the MSC-Exo-miR-181a-5p+NC group to the MSC-Exo-NC+NC group, and further to the MSC-Exo-NC+PTEN group. Correspondingly, cell proliferation in the MSC-Exo-miR-181a-5p+PTEN group was significantly lower than that in the MSC-Exo-miR-181a-5p+NC group (Fig. 4H).

Collectively, these findings demonstrate that PTEN effectively attenuates the promoting effects of exosomal miR-181a-5p on the proliferation, migration, and angiogenesis of HUVECs.

### miR-181a-5p regulates the PI3K/AKT pathway via PTEN

**Fig. 5:**
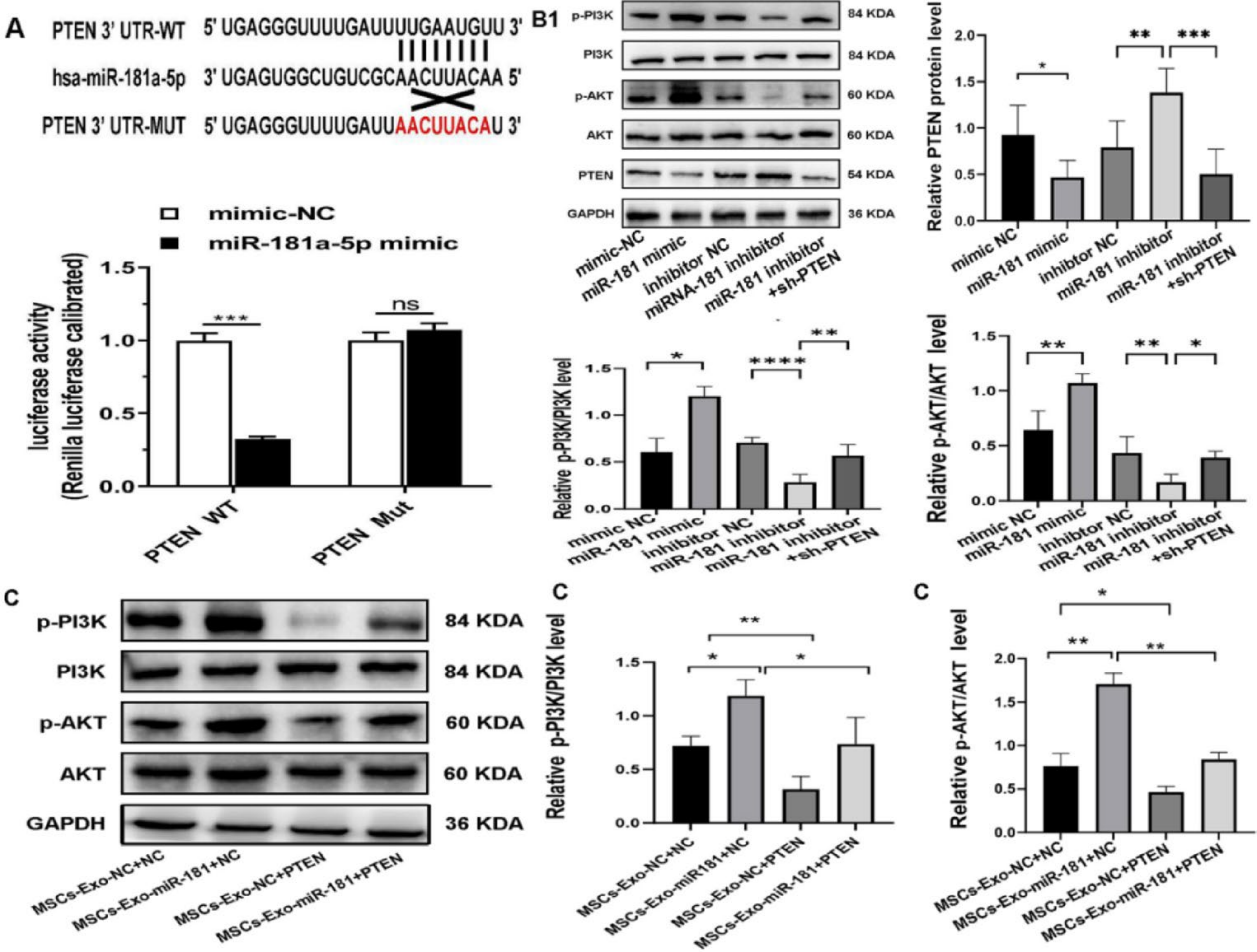
miR-181a-5p regulates PI3K/AKT pathway by mediating PTEN. (**A**) Verification of the interaction between miR-181a-5p and PTEN by dual-luciferase reporter assay; (**B**) PTEN, p-PI3K, and p-AKT protein expression in HUVECs transfected with mimic, inhibitor, or inhibitor+sh-PTEN by western blot analysis; (**C**) Detection of the phosphorylation level of PI3K and AKT by western blot analysis (*p < 0.05; **p < 0.01; ***p < 0.001; ****p < 0.0001).

Our bioinformatics analysis identified the miR-181a-5p/PTEN/PI3K-AKT axis as a key regulatory module. We first confirmed that miR-181a-5p directly targets the 3′UTR of PTEN using a dual-luciferase reporter assay (Fig. 5A).The expression levels of miR-181a-5p, p-AKT, and p-PI3K were consistent, which was in contrast to the expression pattern of PTEN (Fig. 5B).Furthermore, we evaluated the activity of the downstream PI3K/AKT signaling pathway. The expression trends of miR-181a-5p, p-AKT, and p-PI3K were consistent, while all three were inversely correlated with PTEN expression (Fig. 5C).

## Discussion

This study employed an integrated approach combining computational biology with experimental validation to elucidate the pro-angiogenic mechanism of hUMSC-Exos. Preliminary bioinformatics analysis effectively consolidated a complex dataset, identifying the miR-181a-5p/PTEN axis and PI3K-AKT signaling pathway as targets for further investigation. The high concordance between computational predictions and in vitro phenotypic outcomes underscores the biological relevance of our findings and provides a rationale for targeted mechanistic studies in regenerative medicine.

Angiogenesis plays a crucial role in wound healing and tissue regeneration by facilitating the delivery of nutrients and oxygen to injured sites, promoting fibroblast proliferation, collagen synthesis, and re-epithelialization^48,49^. Effective vascular regeneration holds significant importance in regenerative medicine^50^. Angiogenesis serves as a key therapeutic target in both physiological processes (e.g., tissue and organ regeneration) and pathological conditions (e.g.,vascular diseases)^51^. However, achieving functional vascular regeneration in tissue engineering remains a major challenge, with limited vascular regeneration constraining tissue and organ regeneration/repair^52^. The formation of vascular structures is mediated by multiple mechanisms and angiogenic patterns^53^.

MSCs have been shown to enhance diabetic foot wound repair in rats via paracrine effects^54^. Mesenchymal stem cell-derived exosomes are rich in proteins, RNA, cytokines, and growth factors, exhibiting significant potential to promote tissue healing and regeneration^55^. These vesicles retain the biological characteristics of their parent cells, positioning them as a promising cell-free therapeutic approach with broad clinical applicability^51^. Growing evidence indicates that hUMSC-Exos stimulate cell differentiation and promote angiogenesis both in vitro and in vivo^56^. Consistent with these findings, our study demonstrates that hUMSC-Exos promote angiogenesis and proliferation in HUVECs, potentially through activation of the PI3K/AKT signaling pathway. Our previous studies also indicate that MSCs and hUMSC-Exos can be utilized for treating reproductive system disorders^46,47,57^. Collectively, this evidence demonstrates that MSC-Exos represent an effective cell-free treatment for tissue regeneration and reconstruction.

miR-181a-5p is a highly conserved miRNA exhibiting multifunctional roles in vascular homeostasis regulation. It exerts fine-tuned control over the PI3K/AKT pathway by post-transcriptionally silencing key target genes, particularly PTEN, which negatively regulates PI3K signaling. For instance, in breast cancer, hypoxia-induced miR-181a-5p relieves inhibition of the PI3K/AKT pathway by downregulating EPDR1/TRPC1, thereby promoting chemotherapy resistance^58^. Thus, its regulatory role in angiogenesis may involve broader PI3K/AKT-mediated cellular survival signaling networks. Our findings suggest that in our established model, the pro-angiogenic effect of miR-181a-5p is attributable to its suppression of PTEN, which in turn enhances AKT phosphorylation levels and activates key signaling pathways governing cell growth, survival, and vascular remodeling.

PTEN, as a negative regulator of the PI3K/AKT signaling pathway, plays a crucial role in maintaining vascular homeostasis and regulating endothelial cell function^59^. Bioinformatics prediction analysis further suggests that PTEN is a potential direct target of miR-181a-5p. Previous studies have confirmed that miR-181a-5p inhibits PTEN translation by binding to the 3′ untranslated region of PTEN mRNA, thereby releasing inhibition on downstream AKT signaling^60,61^. The miR-181a-5p/PTEN/AKT axis has been established as a critical regulator of multiple cellular processes, including proliferation and angiogenesis^62,63^. Consistent with these reports, our study found through TargetScan prediction and subsequent experimental validation that miR-181a-5p mimics significantly suppressed PTEN expression in HUVECs, accompanied by increased levels of p-AKT^64^. Given the central regulatory role of the PI3K/AKT pathway in angiogenesis, we infer that miR-181a-5p exerts its proangiogenic effects by targeting and modulating the PTEN/PI3K/AKT axis.

High-throughput sequencing and experimental validation confirmed the high expression of miR-181a-5p in hUCMSC-Exos. Previous studies have demonstrated that miR-181a-5p regulates PTEN expression and function, thereby establishing PTEN as its downstream target^34–36^. Therefore, this study focuses on elucidating the linear regulatory relationship among exosomal miR-181a-5p, PTEN, and angiogenesis. Bioinformatics analysis supported the role of miR-181a-5p in regulating PTEN, and subsequent experiments confirmed that HUVEC uptake of exosomes resulted in increased miR-181a-5p and decreased PTEN expression. Notably, the inhibitory effect of exosomes on PTEN was reversed by transfection with a miR-181a-5p inhibitor. Modulation of miR-181a-5p using inhibitors or mimics resulted in corresponding increases or decreases in PTEN expression, accompanied by consistent changes in p-PI3K and p-AKT levels. Cellular phenotype analysis and molecular expression studies, consistent with bioinformatics predictions, collectively indicate that miR-181a-5p from hUCMSC-Exos promotes angiogenesis and proliferation by targeting PTEN.

However, the study has several limitations. First, while our in vitro findings provide strong evidence that miR-181a-5p promotes angiogenesis by regulating the PTEN/PI3K/AKT axis, this mechanism requires further validation in standardized in vivo models. Second, while our study provides indirect support for this regulatory axis via luciferase reporter assays, PTEN rescue experiments, and p-AKT levels, it has not yet employed pathway-specific inhibitors or AKT gene silencing to establish definitive causal relationships. For clinical translation, the following prerequisites are essential: (1) safety assessment in large animal models, (2) development of optimized delivery strategies for exosomal miRNAs, and (3) rigorous efficacy testing in disease-specific settings.

In conclusion, our study demonstrates that hUCMSC-Exos are internalized by HUVECs in vitro. Evidence is provided that hUCMSC-Exos deliver miR-181a-5p, which subsequently downregulates PTEN expression in recipient HUVECs. The miR-181a-5p/PTEN axis is associated with the activation of the PI3K/AKT signaling pathway, correlating with enhanced in vitro tube formation, migration, and proliferation. These findings support a model in which hUCMSC-Exos promote proangiogenic behaviors in HUVECs through the miR-181a-5p/PTEN/PI3K/AKT pathway, at least in part. Further in vivo validation and mechanistic studies are necessary to fully establish the therapeutic potential and the precise contribution of this axis in physiological and pathological angiogenesis.

## Supplementary Information

Below is the link to the electronic supplementary material.

Supplementary Material 1

Supplementary Material 2

Supplementary Material 3

## Data Availability

The datasets used and/or analyzed during the current study are available from the corresponding author on reasonable request.
