# Supplementary material for: miR-181a-5p of MSCs-derived exosomes promote vascular formation and cell proliferation by PTEN/PI3K/AKT axis in HUVECs
Source: Sci Rep. 2026 Apr 16;16:17772. doi: 10.1038/s41598-026-44672-5 (PMC13247093; doi:10.1038/s41598-026-44672-5)

Fig.4B-GAPDH

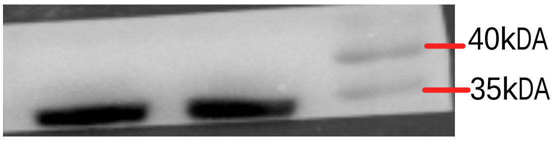

Fig.4B-PTEN

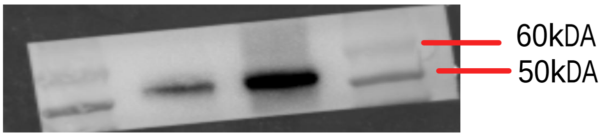

Fig.4D-GAPDH

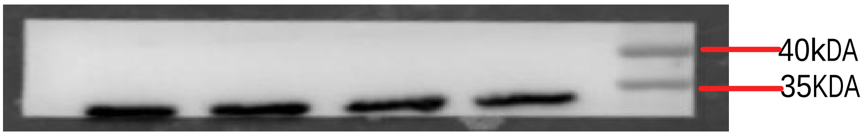

Fig.4D-PTEN

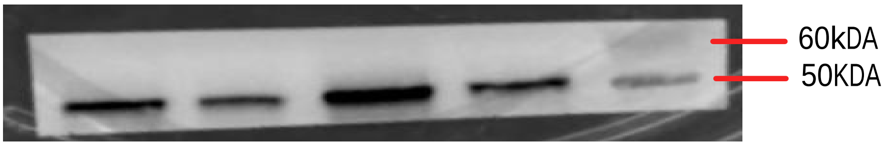

Fig.5B-GAPDH

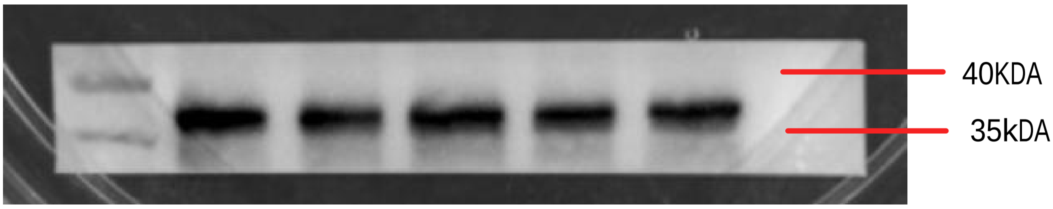

Fig.5B-PTEN

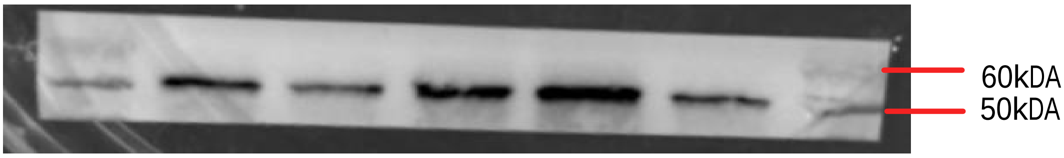

Fig.5B-(p-PI3K)

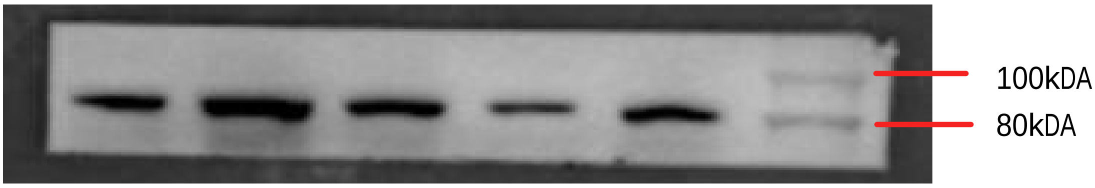

Fig.5B-PI3K

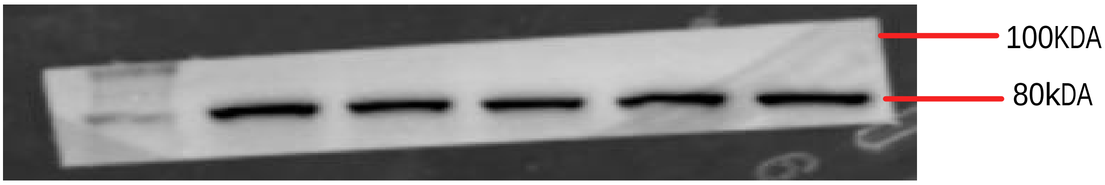

Fig.5B-AKT

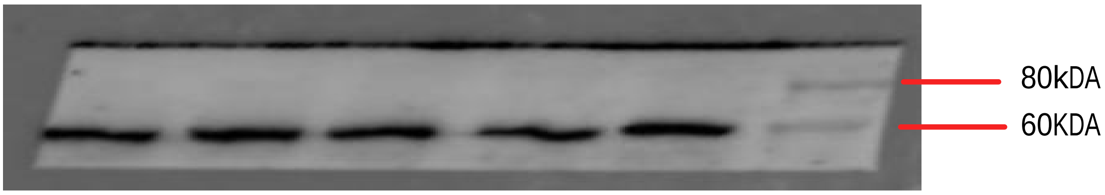

Fig.5B-(P-AKT)

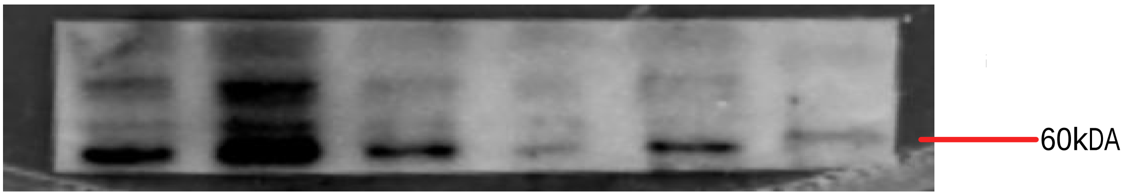

Fig.5D-(p-PI3K)

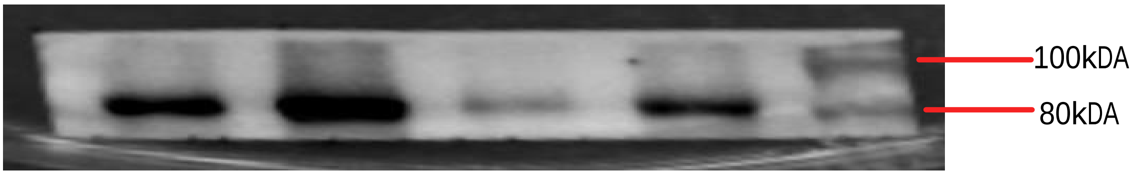

Fig.5D-PI3K

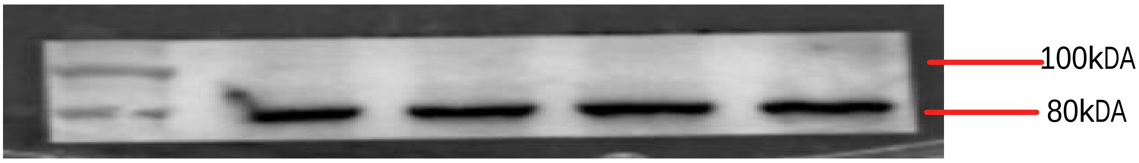

Fig.5D-(p-AKT)

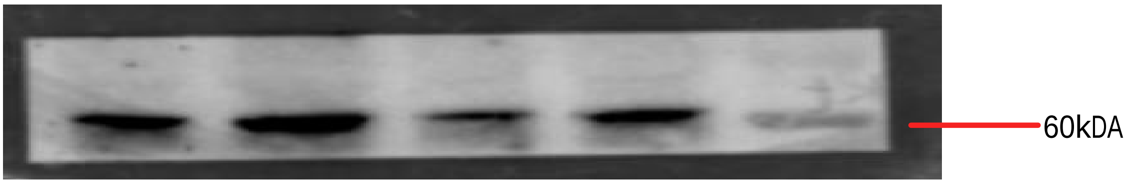

Fig.5D-AKT

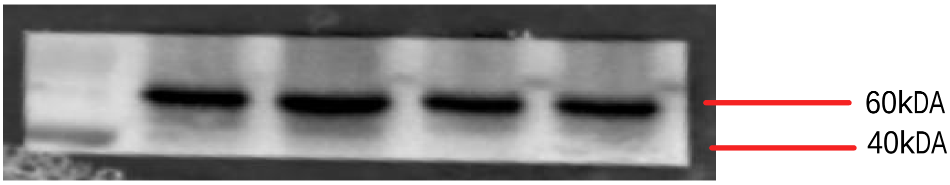

Fig.5D-GAPDH

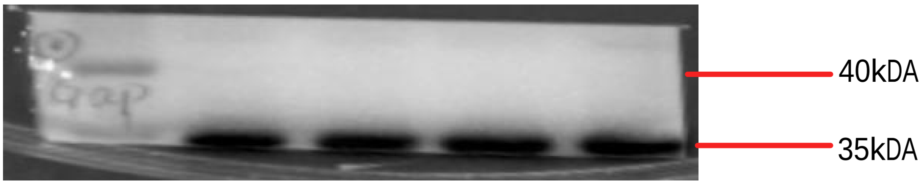

Supplement: Supplementary file 1 — Supplementary Material 1 [file 41598_2026_44672_MOESM1_ESM.pdf]
