# Supplementary material for: miR-181a-5p of MSCs-derived exosomes promote vascular formation and cell proliferation by PTEN/PI3K/AKT axis in HUVECs
Source: Sci Rep. 2026 Apr 16;16:17772. doi: 10.1038/s41598-026-44672-5 (PMC13247093; doi:10.1038/s41598-026-44672-5)
Supplement: Supplementary file 2 — Supplementary Material 2 [file 41598_2026_44672_MOESM2_ESM.pdf]

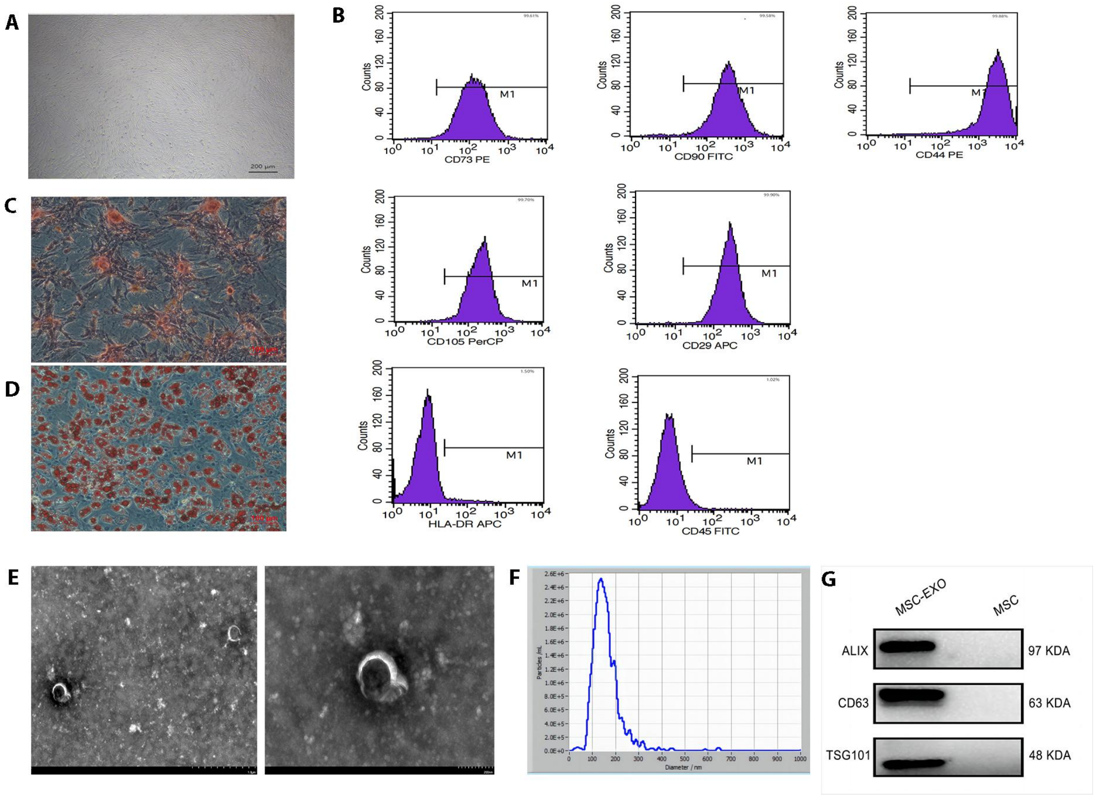

Fig. S1. Characterization and identification of hUCMSCs and hUCMSC-Exos. (A) The morphology of hUCMSCs .Scale bar: 200 mm. (B) hUCMSCs were positive for CD73, CD90, CD44, and CD105,CD29 and were negative for CD45 and HLA-DR, as shown by flow cytometry analysis. (C) Alizarin Red staining was used for hUCMSCs osteogenic identification. (D)Oil Red O staining was conducted for adipogenic differentiation. (E) Representative images of hUCMSC-Exos under transmission electron microscopy. Scale bar: 100 nm. (F) Particle size distribution of hUCMSC-Exos was determined by Flow Nano Analyzer. (G) hUCMSC-Exos were positive for ALIX, CD63 and TSG101,which were shown by western blotting
