# Supplementary figures and images for: miR-181a-5p of MSCs-derived exosomes promote vascular formation and cell proliferation by PTEN/PI3K/AKT axis in HUVECs
Source: Sci Rep. 2026 Apr 16;16:17772. doi: 10.1038/s41598-026-44672-5 (PMC13247093; doi:10.1038/s41598-026-44672-5)

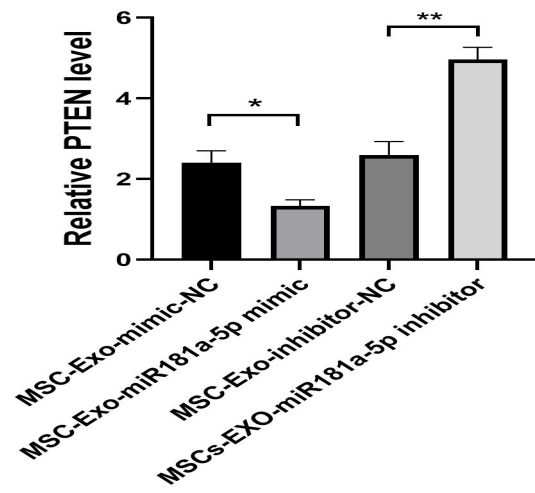

**Figure S2.** The PTEN overexpression/knockdown in HUVECs. (\* $P < 0.05$ , \*\* $P < 0.05$ )

Supplement: Supplementary file 3 — Supplementary Material 3 [file 41598_2026_44672_MOESM3_ESM.pdf]
